# Understanding the excess psychosis risk in ethnic minorities: the impact of structure and identity

**DOI:** 10.1007/s00127-021-02042-8

**Published:** 2021-08-24

**Authors:** Hannah E. Jongsma, Saffron Karlsen, James B. Kirkbride, Peter B. Jones

**Affiliations:** 1 PsyLife Group, Division of Psychiatry, UCL, 6th Floor, Maple House, 149 Tottenham Court Road, London, W1T 7DN UK; 2 Department of Psychiatry, University of Cambridge, Herchel Smith Building, Forvie Site, Robinson Way, Cambridge, CB2 0SZ UK; 3 School of Sociology, Politics and International Studies, University of Bristol, 11 Priory Road, Bristol, BS8 1TU UK; 4 CAMEO, Cambridgeshire and Peterborough NHS Foundation Trust, Elizabeth House, Fulbourn Hospital, Cambridge, CB21 5EF UK; 5 Present Address: Centre for Transcultural Psychiatry ‘Veldzicht’, Ommerweg 67, 7707 AT Balkbrug, The Netherlands

**Keywords:** Psychotic disorders, Ethnicity, Social gradient, Identity, Psychosocial disempowerment

## Abstract

**Purpose:**

Psychotic disorders, which are associated with substantially increased morbidity and mortality, are up to five times more common in some ethnic minority groups compared with the white majority in Western countries. This long-standing and well-replicated public mental health disparity has hitherto largely eluded adequate explanation. We argue that this might have arisen in part due to the lack of attention given to theoretical work characterising the complex and multidimensional social nature of ethnicity by those epidemiological investigations that have dominated the literature.

**Methods:**

To bridge this gap, we draw on theoretical and empirical literature from across the social sciences considering the ontological significance of ethnicity (as biology, migration, racialised structures and identity) and its relationships with psychotic disorders to illuminate probable drivers of excess psychosis risk.

**Results:**

The largest gains in our theoretical understanding of excess psychosis risk among ethnic minority groups are to be made by considering ethnicity in relation to disempowerment resulting from structural and identity-based exclusion. The former is readily studied through the social gradient in health: socioeconomic disadvantage clusters in some ethnic minorities and increases the risk of poor health outcomes, including psychosis. Furthermore, limitations on identity acquisition and expression imposed by the ethnic majority can further contribute to alienate ethnic minorities and increase psychosocial disempowerment (a lack of control over one’s life).

**Conclusion:**

We theorise that structural and identity-based exclusion act as the primary drivers shaping variation in rates of psychotic disorder by ethnic minority status.

## Introduction

Psychotic disorders affect up to three percent of the general population in their lifetime [1] and are associated with a range of poorer health and social outcomes, culminating in an average reduced life-expectancy of around fifteen years [2]. These disorders are up to five times more common in some ethnic minority groups in Western countries. This epidemiological finding is long-established [3], well-replicated [4], and invariant to epidemiological study design [5]. However, this excess risk is not universal across ethnic minority groups. In Cambridgeshire (UK) for instance, the Indian and White non-British ethnic minorities did not face a higher incidence than the White British group [6], and in Australia migrants from South East Asia, China and Southern Asia did not experience a higher risk of psychotic disorders [7].

Progress on identifying the causes of this severe public mental health inequity has remained unacceptably slow. This might have partially arisen because epidemiological studies—which have dominated investigations into this topic—have tended to insufficiently engage with theoretical work on the complex social nature of ethnicity. Two recent reviews attempt to begin to redress this by providing an overview of existing theories and research and discussing the role of structural violence [8], and of various racisms [9]. Our paper seeks to complement and extend these reviews by providing further theoretical consideration to the construct of ethnicity itself and the implications for understanding and studying ethnicity in the context of psychosis risk. While many ethnic minority groups are more likely to be exposed to a variety of social determinants of psychosis, our interest in these risk factors is secondary to our view that to fully understand ethnic disparities in psychosis risk, we need a clear theoretical framework from which to conduct empirical research on this topic. Within epidemiology, ethnicity is often treated as an exposure or a confounder: a fixed category. Whilst such categorisation is somewhat inherent to the discipline, it fails to do justice to the complex social nature in which someone’s experience of their ethnicity arises. Here, we aim to enrich sociological thinking in the epidemiological literature on ethnic disparities in psychosis risk, which has traditionally received little theoretical development. For example, the social defeat theory is frequently cited as a conceptual model for psychosis onset in ethnic minority groups, but only recently has been subject to robust sociological and philosophical analysis [10, 11]. To achieve this, the remainder of this paper will use theoretical and empirical research across the social sciences considering the ontological significance of ethnicity and its relationships with (mental) health, in particular with psychotic disorders.

As will become clear, this research draws on several distinct conceptualisations of ethnicity often implicitly or explicitly invoked in the extant literature to illuminate drivers of ethnic inequalities in health, and specifically psychosis risk. We do not believe all of these conceptualisations are equally helpful or valid and acknowledge there are many other conceptualisations of ethnicity (such as viewing ethnicity as culture or through a historical lens). In turn, this paper will review evidence for the impact on the psychosis of ethnicity as biology, ethnicity as migration and links to ancestral environments, ethnicity as structure and ethnicity as identity. Our overall aim in this paper is to enhance understanding of psychosis through a study of these various conceptualisations of ethnicity, and of ethnicity and health using psychosis as a case study.

## Ethnicity as biology

There is no reason to assume that there is any biological or genetic cause of the excess risk of psychotic disorders in certain ethnic minority groups. Most of what is known about the genetics of psychotic disorders, for instance, is limited to schizophrenia. Here most of the genetic variation in risk as of yet unexplained [12] and what little has been explained is highly polygenic: many small mutations each carrying a very small excess risk. To date, most genetic studies have been carried out predominantly in white groups, and results are not considered directly translatable to people of different ethnic origins due to systematic differences in genetic ancestry [13]. Such differences are small, but sufficient to limit generalisability of genetic markers of a disorder.

More importantly, there is, in fact, strong evidence against a genetic explanation of psychosis risk from studies where the same ethnic group have a very different incidence of psychotic disorders, depending on where they live. For example, in Ødegaard’s seminal study of Norwegian migrants in Minnesota rates of schizophrenia were higher in emigrants than they were in the general population in Norway [3]. In Trinidad [14], Surinam [15] and Jamaica [16] rates of schizophrenia have been found to be much lower than rates of psychosis estimated using similar methodology in minority populations in Western Europe originating from these countries. In fact, rates in the general population in Trinidad, Surinam and Jamaica were comparable with rates in the white majority in Western European countries [14, 15]. More recent tentative evidence from Nigeria, Trinidad and India suggests that the burden of any psychotic disorders (schizophrenia, as well as other psychotic disorders) in different low-and middle-income countries might be substantial, though these studies are not be directly comparable due to differences in case-finding methodology and diagnostic criteria [19]. While this remains an area for future investigation, at present there is no substantial evidence that some ethnic groups are ‘inherently’ more at risk of psychotic disorders.

Any excess risk in broad ethnic minority groups appears to not only differ by majority/minority status but also appears to differ by country of residence. For example, in England, the risk of people of Black Caribbean descent is higher than that of any other ethnic group, with a pooled risk of 5.6 times higher than the general population [18]. Yet in the Netherlands, while people of Black Caribbean origin (from the Dutch Antilles) are at increased risk, this is smaller: 2–3 times that of the general population [17]. Indeed, in the Netherlands, Moroccan immigrants face the highest increase in risk with incidence rate ratios (IRRs) between 4 and 6 [17], whereas in France, the North African minority appears not to face an excess risk [20].

The lack of biological basis for this health inequity does not mean there is nothing to be gained from a biomedical or biopsychosocial approach to understanding the aetiology of mental disorders, including psychosis. Such an approach is crucial for understanding how the environmental adversities described in later sections of this paper get biologically embedded and leads to increased risk of disorder. This will be discussed after examining various other conceptualisations of ethnicity.

## Ethnicity as migration

A common understanding of ethnicity is through links with ancestral ‘homelands’. This has translated to a focus on migrant status, and conceptualisation of psychosis risk in relation to pre- and post-migratory circumstances and issues related to migration itself. Viewing ethnicity purely in relation to migration is erroneous: many individuals from an ethnic minority background have never migrated and can thus not be considered migrants. However, a historical focus on ethnicity as migration has improved our understanding of psychosis in migrants and has led to the identification of high-risk groups, such as refugees [21], and as such we will review this conceptualisation here.

An often-suggested, but poorly supported hypothesis for excess psychosis risks in some ethnic minorities, in line with viewing ethnicity as migration, is the so-called ‘unhealthy migrant’ effect, whereby it was suggested that those already more vulnerable to developing psychosis were more likely to migrate [3]. This selection hypothesis appears to be an implausible mechanism: migrants’ physical and mental health, at least initially, appears to be at least as good as the general population health in the countries to which they move [22]. Of specific relevance to psychosis, an epidemiological thought experiment on the excess psychosis risk of the Surinamese minority in the Netherlands showed that even if the entire population of Surinam would migrate to the Netherlands (trebling the denominator) and would not contribute any extra cases, the Surinamese population in the Netherlands still faced an increased incidence of schizophrenia [23].

While there is evidence that pre-migratory exposure to stressors and the process of migration itself contribute to excess risk in psychotic disorders in migrants (as evidenced by the particularly high rates of the disorder among refugees [21]), it is also clear that, despite never having migrated, excess psychosis risk persists in the descendants of migrants [4]. Given these observations, conceptualisations of ethnicity as migration may be less useful in contemporary Western European countries where an increasingly large proportion of people who identify as belonging to an ethnic minority group are not migrants. Conceptualising ethnicity as migration also leaves no room to examine any excess risk of psychotic disorders in indigenous minorities in predominantly white countries and of long-standing minorities such as African-Americans. This is significant given that these minorities often experience similar levels of structural and cultural marginalisation and face worse mental health outcomes, including a higher risk of psychotic disorders, than the white majority population [24–27]. This conceptualisation of ethnicity also is insufficient for countries which are very ethnically heterogeneous, but where a white minority might hold a disproportionate share of the economic and political power and where ethnic minorities might still face an excess psychosis risk, such as South Africa and Brazil [28].

Ultimately, whilst viewing ethnicity as migration may remain a relevant conceptualisation while global migration continues to increase, it is ultimately too limited a view of ethnicity and is insufficient to explain the present ethnic patterning of psychosis identified in Western countries.

## Ethnicity as structure

Ethnicity can also be understood as an axis through which social structures become manifest, whereby ethnic collectives enjoy differential access to a variety of social resources [29]. In other words: access to social and economic opportunities might be more limited for those from some ethnic minority backgrounds due to the inequitable social structures which become established, maintained and reinforced over time. Seen through this lens, we would expect ethnic minority status to be associated with psychosis risk through a social gradient in health: the better off people are, economically and socially, the better their health [30]. This social gradient in health has been widely researched in the context of mortality [31], cardiovascular disease [32] and, to a lesser extent, common mental disorders [33]. We argue that this paradigm is also apt for researching psychotic disorders, although explicit studies into its role in psychosis onset remain limited [34]. In this section, we will outline evidence pertaining to ethnicity as structure, as a disadvantage is often strongly patterned by ethnicity, and in a later section we will focus our attention on the mechanisms through which this could increase psychosis risk. While a social gradient in health exists within ethnic groups [35], certain ethnic minorities are also concentrated in lower socioeconomic positions which could explain their greater risk of physical and mental ill-health. Understanding ethnicity in the context of these racialised social (power) structures, therefore, requires an intersectional analysis [36], as separate analyses of minority status and poverty or socioeconomic disadvantage do not do justice to the full accumulation of disadvantage experienced by ethnic minority groups [37].

The 2012 Poverty and Social Exclusion survey in the UK demonstrated that several ethnic minority groups were more likely to live in poverty than the white British majority, predominantly the Black African, Black Caribbean, Bangladeshi, Pakistani and Polish groups [38] and that poverty was also more likely to be persistent across these various groups. This echoes data from the 2011 Census, which showed that employment was lowest in the Pakistani and Bangladeshi groups [39], and that over half of all households from the Bangladeshi, other Asian and Black ethnic groups fell into the two lowest income quintiles [40]. Similarly, in The Netherlands, 5.6% of households where the main breadwinner was Dutch lived in poverty in 2016, compared with 10.1% of households where the main breadwinner was from a Western migration background, and 26.3% of households where the main breadwinner was from a non-Western migration background [41]. This disadvantage is not limited to income: ethnic minorities are more likely to only have attended primary school in the Netherlands (14.3%, versus 10.1% of white Dutch people [42]), while only 36.8% of the Hispanic group in the USA had some college education (versus 63.8% of non-Hispanic Whites [43]). Just as not all ethnic minorities face excess psychosis risk, not all minority groups experience higher levels of socioeconomic disadvantage. For example, in the UK, men of Indian and white other ethnicities are employed at a higher percentage than White British men (83 and 88 vs 80%) [39].

Any investigation into social determinants of psychosis is hampered by the validity of standard measures of socioeconomic exposures. These are designed with reference to the white majority population, and their validity appears limited across ethnic minority groups—consequently their use can disguise significant inequality between groups [44]. A persistent ethnic penalty affects access to employment, education and other aspects of social position: for the same level of resources and capabilities, individuals from ethnic minority backgrounds yield a lower return [45, 46]. For example, any given level of education does not open up the same economic opportunities for those with minority ethnicities as it does for the white majority [47]. The use of socioeconomic markers based on occupations class can also obfuscate differences in income. Indeed, the ethnic minority pay gap can be so large that some Pakistani workers in the highest social class earn less than white British workers in the lowest [47]. The value of such measures of social class to establish the relative economic position of minorities is, therefore, severely limited [47], hampered by the differential measurement error described in this paragraph.

Moreover, socioeconomic disadvantage is not merely a confounder of the relationship between ethnicity and psychosis risk, as often considered [6, 48], but is on the causal pathway between ethnic minority status and psychotic disorder: minorities are more likely to be disadvantaged, and this disadvantage contributes to health inequities. Epidemiological research in this field has, thus far, failed to correctly model such associations. This relationship between ethnicity and social disadvantage not only affects minorities’ probability of experiencing stressors (as discussed above) but also increases vulnerability to their adverse health effects. Individual characteristics such as control, self-efficacy, trust and resilience can help mitigate the negative effects of disadvantage [49], but are in themselves also subject to a social gradient: access to them is limited for those who are disadvantaged [49].

The measurement inadequacies affecting analyses seeking to adjust for socioeconomic variation between ethnic groups might explain the excess health risk which remains following adjustment for socioeconomic factors [34, 50]. However, we instead argue that viewing ethnicity purely through the lens of socioeconomic disadvantage cannot fully explain excess psychosis risk in ethnic minority groups, even if socioeconomic disadvantage were to be measured perfectly. Ethnicity seen purely through a structural lens ignores the fundamentally interpersonal nature of ethnicity, as inherent to our social identity. These identities can be constructed to the benefit of one group, but at the detriment of others; thus, ethnicity as identity may offer a powerful mechanism for understanding the links between ethnic minority status and psychotic disorders. These challenges are detailed in the next section.

## Ethnicity as identity

Identity is understood to be an affective claim to identify a human collective to which we feel a sense of belonging [29]. Identity is essentially concerned with who we are in relation to others [51]. This is important for wellbeing: people derive utility or value from expressing their identity in a way that they believe is in line with what they, and the human collective they’re part of, expect of them [52]. Identity is not only important at the individual level. A shared sense of identity is important for societies in terms of the trust that supports social cooperation, the empathy that supports financial and other redistribution and people’s willingness to contribute to public goods [53]. Everyone also exhibits multiple identities (related to the migrant status, gender, age, sexual orientation, education, political orientation, religious beliefs and so forth), and their relative importance is influenced by other aspects of identity. Our identities are also fluid, over time and across social circumstances [29, 54, 55]. Identity formation and expression are also of direct relevance to the development of psychotic disorders. Humans have a basic psychological need for social connectedness and a sense of belonging [56]. Psychosis is characterised, in essence, by an increasing disconnect with the outside world resulting from and contributing to a reduced sense of belonging. This has a complex, disruptive effects on one’s sense of self, and on identity [57]: identities that were previously accessible or important are now closed or diminished and new ones are superimposed (‘someone with a psychotic disorder’). Any threat to identity, group membership and belonging is, therefore, an important potential contributor to psychosis risk.

Our identities give us a sense of who we are and codify our behaviours in various circumstances [52]. We form our identities and norms of behaviour associated with them on the basis of our past choices [58], and, importantly, on the basis of our social context [52]. Forming an identity is facilitated by complex forms of behavioural inference, imitation and anticipation [59]. They are fundamentally social processes and consequently crucially dependent on others. This identity formation, as well as its maintenance and expression is more complicated for (ethnic) minorities due to inequalities in the balance of power in society. Which identities ethnic minority individuals can exhibit, the borders of these identities and which behavioural and social norms are acceptable, are less autonomous for minority groups and foisted upon them by the majority population [42, 49].

There can be no singular ‘ethnic minority’, identity. Yet, these societal power imbalances mean that often groups become characterised solely according to certain conceptions of their ethnic identity held by the ethnic majority: an ‘unfamiliarity homogeneity effect’ where everything that is unencountered and unfamiliar becomes uniform [60]. This leads to individuals more able to recognise heterogeneity as well as potential bonds of commonality among members of what they perceive to be their own group. Ethnicity is often used to create or exaggerate perceived difference [36] by those in power.

There are two main direct consequences of this denial of the plurality and fluidity of ethnic minority individuals’ identities: stereotype threat and exclusion. Stereotype threat results from the negative portrayal of ethnic minorities in media and popular culture and describes the situation where an individual is concerned about being judged on the basis of such a negative stereotype [61]. This can have far-reaching consequences. For example, it is often invoked as an explanation of the underperformance of minorities such as African-Americans on standardised tests [62]. ‘Insider’ status can also be made implicitly contingent on holding ethnic majority status, leaving minorities permanently excluded from a range of economic, social and political opportunities [52]. The discrimination associated with such prejudice is an important explanation for the ethnic penalty described earlier, where despite equivalent educational qualifications economic opportunities remained more limited to ethnic minorities.

The socioeconomic implications of such singular and often stereotypic views of ethnic (minority) identities held by the ethnic majority contribute to the overall exclusion and subordination faced by many ethnic minority groups in Western societies. This exclusion and subordination, in turn, leads to a sense of psychosocial disempowerment: not being in control of your life. This disrupts a sense of self, which helps to navigate various identities in situations where they might conflict [63]. Such disempowerment can be experienced in an awareness of having difficulties to achieve your goals, including overcoming the barriers imposed on racialised groups by wider society. Consciously experiencing a lack of control over your life could be considered the extreme end of psychosocial disempowerment. Such an external locus of control attributes negative events to external causes, and these and other deficits in social cognition are associated with psychosis [64].

This subjective experience of exclusion from the majority population is considered by some to be the cause of excess psychosis risk in ethnic minorities [65]. Yet, the obstacles to success faced by ethnic minorities are very real, and understanding ethnicity through the lens of racialised power structures alongside ethnicity as identity can greatly improve our insight into the impact of this accumulated disadvantage and exclusion on psychotic disorders.

## Biological embedding

There remains a gap in understanding regarding the impact of the social gradient, identity and psychosocial disempowerment on psychosis. There appear to be two plausible mechanisms affecting the translation of the dual environmental adversities of socioeconomic disadvantage and identity-based exclusion detailed in this paper into an increased risk of experiencing psychotic disorders: biological embedding and neuroscience. Research into the detrimental effects of low socioeconomic status emphasises the psychosocial pathways through which social factors affect the mind [49] or the ways in which social risk factors get ‘under the skin’ [66]. Much attention in the social gradient literature has been given to the consequences of experiencing a lack of control over one’s life, or psychosocial disempowerment [67]. This is posited to be biologically embedded through a chronic ‘fight or flight’ response [68] or allostatic load, where hormones that protect the body and promote adaptation in the short term are associated with negative changes in the brain and body in the long term. One of the core elements of the stress response system is the hypothalamic–pituitary–adrenal (HPA) axis [55, 57]. Arousal of this system both leads to behavioural, cognitive and physiological responses to environmental stressors and induces further arousal in a range of central and peripheral areas of the nervous system [68], including areas implicated in the onset of psychotic disorders.

A more neuroscientific understanding of this translation from the environment to psychosis draws on the tradition of social psychiatry, which considers psychotic disorders as disorders of social functioning [69] and understands mental illness more broadly in relation to an individual’s social context. This has been conceptualised in many ways, but one attractive, unifying theory of psychosis is that of predictive processing. This theory holds that the brain is essentially a predictive processor, which under typical circumstances makes thousands of computational predictions about expected outcomes given environment and prior knowledge. When a mismatch occurs between what is expected and what actually transpires—a prediction error—we recalibrate our computational models based on the size of the prediction error and the trustworthiness of its source [70].

Stereotype threat and identity-based exclusion contribute to unrealised expectations and ambitions, as well as psychosocial disempowerment. It is less likely that people experiencing this are able to freely express a variety of identities and it becomes more difficult to assess which identity is acceptable in which situation. This makes for a more volatile (or even hostile) environment. This volatility means that in this particular environment, there are higher levels of uncertainty associated with forming your anticipated interactions with your environment and that it is more difficult to interpret whether or not any given interaction was as expected. Thus, being exposed to such volatility increases the probability and magnitude of a mismatch or prediction error. Not knowing what to expect or how to interpret what is experiences also makes it harder to assess the salience of these prediction errors. Over-interpreting salience in recalibration may lead to false inferences about the real word, manifested as delusions or hallucinations.

## Outstanding issues

In this paper, we have explicitly discussed a number of conceptualisations of ethnicity that have been frequently implied in epidemiological literature on excess psychosis risk in ethnic minorities. This serves as the start of a more active engagement of epidemiology with the social sciences on this topic. That notwithstanding, an important conceptualisation of ethnicity that was not explicitly discussed in this paper is of ethnicity as culture, where ethnicity is primarily viewed as being determined by a set of ideas, values and understandings used to order interpersonal relationships and give meaning and purpose to live [71]. Such a conceptualisation of ethnicity is sometimes invoked to understand the more negative pathways to care experienced by ethnic minorities (i.e. more frequently via contact with the police), excessive use of detention and poorer outcomes after therapeutic and medical interventions [72]. These are different research questions and not the subject of this paper. As with increased psychosis risk in ethnic minority groups, poorer outcomes or higher mortality following a diagnosis are also not universally established [73]. Furthermore, invoking cultural explanations (through for instance a focus on acculturation) risk focussing too much on individual values and behaviours and obscuring the role of structural determinants of health inequities [74]. We also did not explicitly discuss further conceptualisations of ethnicity (for instance through a historical perspective) or the important issue of interpersonal (direct) racism; this paper has been largely concerned with indirect racism.

A further limitation is that the framework presented in this paper risks homogenising what is a heterogeneous experience both within and between particular ethnic groups. Among each ethnic minority group in each country, there will be a unique interaction of risk and protective factors. Whilst there will be overlap in these factors, inconsistencies in risk patterns caution us against considering the work put forward in this paper as a unifying theory of excess psychosis risk in ethnic minorities.

Further theoretical and empirical research must be conducted in tandem to assess the applicability of this framework to the experience of particular individuals and groups. In particular, we suggest that greater work is required to unpack the role of identity, ethnicity and psychosis risk. Nonetheless, solely focussing on ethnicity as an identity also risks inadvertently contributing to othering ethnic minorities and falling foul to the categorical essentialism we have described. By zooming in on one element of a complex, multifaceted and fluid identity, we ascribe an importance to it that will not be shared by everyone, and we assume a commonality of experiences of identity that is unlikely to be true. Our paper has also not given adequate attention to the implications of our proposed framework by other intersectional axes such as generational status or for individuals of mixed ethnicity. Nor have we explored the relative importance of ethnicity vis-à-vis other identity categories in contributing to patterns of psychosis risk in the population. That said, as we suggest, part of the explanations for these ethnic inequalities in psychosis may be just this: the implications of the blindness to difference caused by racializing stereotypes leading to greater homogeneity of experience than is to be reasonable expected given our diverse and fluid identities.

It is also likely that a better understanding of the mental health impact of structural disadvantage and othering and exclusion as a result of identity is also of value for those in other minority groups (e.g. religious minorities, the LGBTQI  + community). There is an emerging literature on the poor mental health of sexual minorities [75, 76], but limited research into psychotic disorders specifically and into drivers of this, although this is also likely to be affected by experiences of symbolic (and actual) violence.

Understanding of the impact of ethnicity as identity in the context of psychosis remains limited. This is perhaps in part due to the issues associated with quantifying these effects using routinely collected data in a field dominated by survey research. Operationalising the required intersectional frameworks in quantitative research remains a challenge. We [34], and others [37], have attempted this in the context of ethnic minority mental health. This has provided a more detailed understanding of the complexity of drivers of excess morbidity in various minority groups. There is a rich sociological literature on ethnicity, race, identity and intersectionality, and integrating this into epidemiological literature and practice requires interdisciplinary collaborations [77].

Finally, more theoretical and empirical work needs to be done to establish the falsifiability of this framework. This should include investigation of how this framework fits in with the wider literature on psychosis risk factors, the exact psychosocial and neurobiological pathways from structural disadvantage, identity-based exclusion and psychosocial disempowerment which contribute psychosis and on any insights into preventative action that this framework might offer. More work is also needed to understand how entrenched, systematic and racialised biases in society contribute to psychosis, including prevailing political climates, colonial legacies and attitudes toward minoritised groups.

## Conclusion

There will be no single, simple explanation for the higher rates of psychotic disorders observed in some ethnic minority groups. Ethnicity is a social phenomenon and the experience of any particular ‘ethnicity’ will be heterogeneous. As such, it is perhaps not surprising that ethnicity does not map neatly and consistently onto particular patterns of psychosis. In this paper, we aimed to improve understanding of this heterogeneity by viewing the concept of ethnicity in different ways. Whilst there is no basis for understanding either ethnicity or ethnic differences in psychosis risk as genetically or biologically determined, we need a biomedical approach to understand how environmental adversity can get ‘under the skin’. Understanding ethnicity in relation to ancestral homelands and consequently mainly in the context of migration has led to the identification of high-risk groups such as refugees. It has thus not only improved our understanding of aetiology but also identified concrete targets for public health interventions such as increased psychological support for refugees. Yet this conceptualisation of ethnicity is insufficient both in the capture of minority groups and to explain the excess psychosis risk in other groups in contemporary Western societies.

The best opportunity to progress understanding of this excess risk of psychosis is through an understanding ethnicity as structure and as identity. Ethnicity as the structure can be understood in terms of the ways in which social adversity can accumulate to negatively affect the psychosis risk of those with certain ethnicities. However, ethnicity is also a fundamentally social construct and perceptions of ethnic identity are readily used as a way of excluding minorities from attaining implicitly racialised identities and privileged positions in society. These dual adversities of accumulated disadvantage and exclusion can lead to increased psychosocial disempowerment among minority groups, which contributes to an increased risk of psychotic disorders through biological embedding and predictive processing. Understanding the complex interplay between these elements is crucial for to enable us to pave the way for preventative interventions addressing this important public mental health inequity and reducing these ethnic inequalities in psychosis risk.
